# Systematic Review of Nutritional Guidelines for the Management of Gestational Diabetes Mellitus: A Global Comparison

**DOI:** 10.3390/nu17142356

**Published:** 2025-07-18

**Authors:** Angelo Sirico, Maria Giovanna Vastarella, Eleonora Ruggiero, Luigi Cobellis

**Affiliations:** Department of Woman, Child, and General and Specialized Surgery, University of Campania “Luigi Vanvitelli”, 80138 Naples, Italy; mariagiovannavastarella@hotmail.it (M.G.V.); ele.ruggi93@gmail.com (E.R.); luigi.cobellis@unicampania.it (L.C.)

**Keywords:** gestational diabetes, pregnancy, nutrition, review, guidelines, macronutrients, micronutrients

## Abstract

**Background:** Gestational diabetes mellitus (GDM) affects 7–9% of pregnancies worldwide and is associated with adverse maternal and neonatal outcomes. Nutritional therapy is a key component of GDM management. However, inconsistencies exist across international and national guidelines regarding macronutrient distribution, glycemic targets, and micronutrient supplementation. This systematic review aims to compare updated nutritional recommendations for GDM across major health organizations and identify areas of consensus, divergence, and evidence gaps. **Methods:** This systematic review was conducted following PRISMA guidelines and registered in PROSPERO (CRD420251026194). A comprehensive literature search was performed in PubMed, Scopus, and Google Scholar (concluding March 2025), along with manual searches of official websites of professional health organizations (e.g., ADA, WHO, NICE, IDF). Guidelines published within the last 10 years (or the most relevant national guideline if slightly older), available in English or with access to translation, and including explicit nutritional recommendations for GDM were included. Data were extracted on macronutrient composition, glycemic targets, and micronutrient supplementation, with evaluation of the supporting evidence and regional context, incorporating findings from recent key guideline updates. **Results:** In total, 12 guidelines met the inclusion criteria. While all guidelines emphasized carbohydrate moderation and adequate fiber intake, significant discrepancies were found in carbohydrate quality recommendations (e.g., low-glycemic index focus vs. total carbohydrate restriction), postprandial glucose targets (e.g., 1-h vs. 2-h measurements and varying thresholds like <120 vs. <140 mg/dL), and the use of non-routine micronutrients such as chromium, selenium, and omega-3 fatty acids (generally lacking endorsement). Recent updates from key bodies like ADA, Diabetes Canada, and KDA largely maintain these core stances but show increasing emphasis on dietary patterns and acknowledgement of CGM technology, without resolving key discrepancies. Cultural adaptability and behavioral counselling strategies were minimally addressed across most guidelines. **Conclusions:** Despite general agreement on the principal recommendations of nutritional management in GDM, substantial variation persists in specific recommendations, even considering recent updates. Consistent, evidence-based, and culturally adaptable guidelines incorporating implementation strategies are needed to optimize care and reduce disparities in GDM management across regions.

## 1. Introduction

Gestational diabetes mellitus (GDM) is a prevalent condition affecting approximately 7–9% of pregnancies globally, with its incidence rising due to increasing rates of obesity and sedentary lifestyles [1]. GDM is associated with several maternal and fetal complications, including preeclampsia, macrosomia, neonatal hypoglycemia, and the long-term development of type 2 diabetes mellitus (T2DM) for both mother and child [2]. In particular, GDM has been demonstrated to affect placental development, leading to changes in neoangiogenesis and inflammation markers, and fetal cardiac function, with changes in parameters such as fetal heart rate and myocardial performance index compared to pregnancies without GDM [3,4,5,6].

The management of this condition has become a critical public health priority with nutritional therapy still considered a cornerstone in managing GDM, aiming to achieve glycemic control, ensure the health of the mother and fetus, and reduce the risk of complications [7].

Despite the importance of nutrition in managing GDM, there is a considerable lack of standardization in the guidelines provided by various global and national health organizations and variations exist in key aspects of nutritional therapy, including macronutrient distribution, glycemic targets, and the recommendation for micronutrient supplementation. For instance, postprandial glucose targets vary widely, with some guidelines recommending a 1-h measurement (<140 mg/dL) while others focus on a 2-h target (<120 mg/dL), leading to different management intensities. These differences are influenced by factors such as regional dietary habits, cultural norms, available healthcare resources, and interpretations of evolving scientific evidence. This variation is associated with a lack of uniformity in the clinical management of GDM and contributes to uncertainty in practice. Therefore, the purpose of this review is to provide a comprehensive comparison of international and national guidelines on the nutritional management of GDM, focusing on macronutrient recommendations, glycemic targets, and the role of micronutrient supplementation. By examining these guidelines, this review seeks to identify areas of consensus, divergence, and potential gaps in evidence that need to be addressed in future research.

## 2. Materials and Methods

This systematic review followed the Preferred Reporting Items for Systematic Reviews and Meta-Analyses (PRISMA) guidelines and was registered in PROSPERO (CRD420251026194) to ensure methodological rigor and transparency [8,9,10] (Tables S1 and S2). We aimed to evaluate and compare the dietary recommendations for GDM management across a range of international and national professional guidelines, analyzing the evidence supporting these recommendations.

A comprehensive search was performed in PubMed, Scopus, the Cochrane Library, and Google Scholar (concluding March 2025) using keywords such as “gestational diabetes nutrition guidelines,” “dietary management of GDM,” and “nutritional recommendations for GDM.” We also reviewed the official websites of major health organizations, including the American Diabetes Association (ADA), the International Diabetes Federation (IDF), the World Health Organization (WHO), Diabetes Canada, the National Institute for Health and Care Excellence (NICE), and the Asia-Pacific Diabetes Federation (APDF), among others.

Inclusion criteria for the review required that guidelines be published in the last 10 years (relative to the search conclusion date), available in English or with accessible translations, and include clear recommendations on nutrition for GDM management. Documents without explicit recommendations or those older than 10 years were generally excluded; however, the 2010 French guideline [11] was included as it remains the most relevant national guideline available for France and is frequently cited as a key European comparator. Two independent reviewers (A.S., M.G.V.) screened the documents and discrepancies were resolved through consensus. Data were extracted on three primary areas, macronutrient distribution, glycemic targets, and micronutrient supplementation, with particular attention to the evidence level supporting each recommendation. Where available and relevant to key points of comparison, information from the most recent updates of major included guidelines (specifically ADA 2024 [12], Diabetes Canada 2023 [13], KDA 2023 [11]) was incorporated into the results and discussion to reflect the current state.

We also examined the frequency of guideline updates to assess whether recommendations have evolved over time. Furthermore, we analyzed the inclusion of patient-centered approaches such as behavioral counselling and adherence strategies, which may enhance the effectiveness of dietary interventions. The quality of the supporting evidence was assessed, distinguishing between high-quality clinical trials, observational studies, and expert consensus. This classification helped contextualize the strength of the recommendations provided by each guideline. In particular, to formally assess the methodological rigor and transparency of the included guidelines, two independent reviewers (A.S., M.G.V.) conducted a quality appraisal using the Appraisal of Guidelines for REsearch & Evaluation II (AGREE II) instrument [14]. The AGREE II tool consists of 23 items organized into six domains: (1) Scope and Purpose, (2) Stakeholder Involvement, (3) Rigor of Development, (4) Clarity of Presentation, (5) Applicability, and (6) Editorial Independence. Each item was scored on a seven-point scale and domain scores were calculated as a percentage of the maximum possible score. Discrepancies in scoring were resolved by consensus. We performed the analyses using Microsoft Excel

## 3. Results

**Figure 1:**
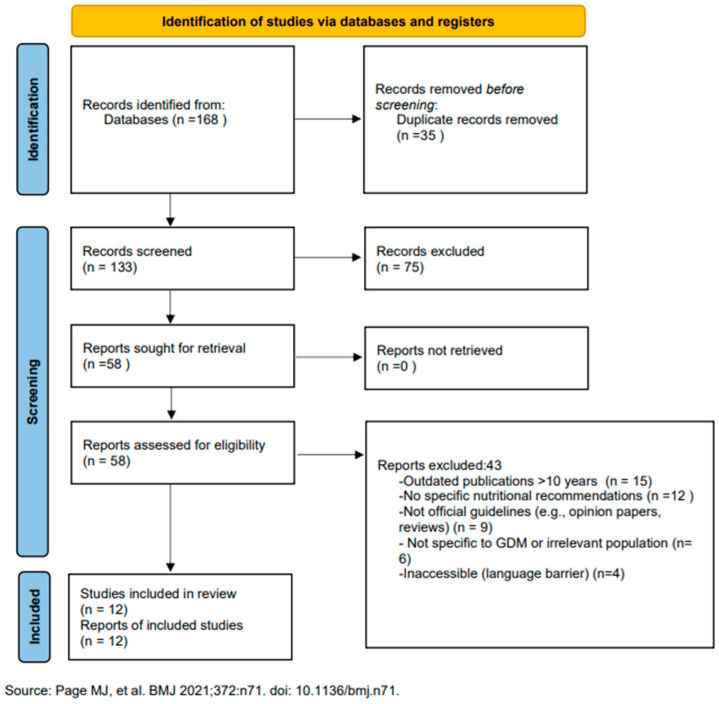
PRISMA 2020 flow diagram illustrating the identification and selection of included guidelines [9].

From 168 initially identified records, 58 full-text guidelines were reviewed in detail and 12 met all inclusion criteria for the primary analysis (see PRISMA flowchart in Figure 1).

**Figure 2:**
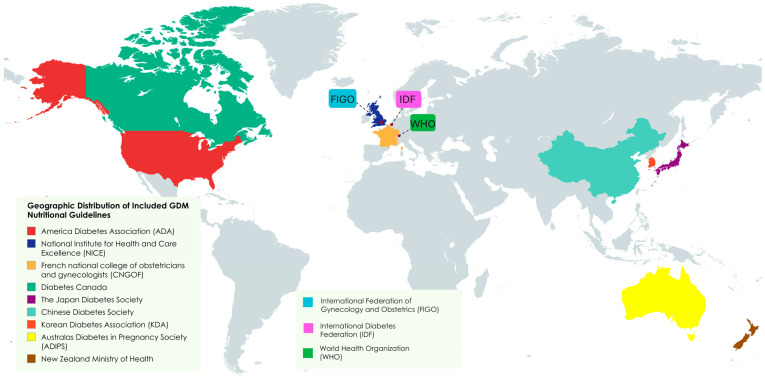
Geographic distribution of included nutritional guidelines for GDM pregnancies.

These included guidelines from North America (ADA [12], Diabetes Canada [13]), Europe (NICE [15], French CNGOF/SFD [16]), Asia (Japan [17], China [18], South Korea [11]), Oceania (Australia/New Zealand [19,20]), and international bodies (WHO [21], IDF [22], FIGO [23]) Figure 2.

The methodological quality of the guidelines was formally assessed using the AGREE II instrument. The IDF Diabetes Atlas was excluded from this appraisal as it is an epidemiological report, not a clinical practice guideline. The results, summarized in Table 1, reveal considerable variability in quality across the documents. Overall, guidelines from NICE, New Zealand, WHO, ADA, Japan, Korea, and Canada demonstrated good to excellent methodological rigor, particularly in the domains of “Rigor of Development” and “Clarity of Presentation.” In contrast, guidelines from FIGO, China, France, and Australia (ADIPS) scored lower, especially in the “Rigor of Development” domain, indicating a greater reliance on expert consensus rather than a formal systematic evidence review process. “Stakeholder Involvement” and “Applicability” were domains with lower scores across most guidelines, with the New Zealand guideline being a notable exception for its high performance in these areas. “Clarity of Presentation” was a universal strength, while “Editorial Independence” was generally well-reported.

When analyzing the included guidelines from different health organizations, the overall findings demonstrate significant alignment in certain areas of GDM management, as well as notable discrepancies in others. Across the 12 guidelines analyzed, 100% (*n* = 12) endorsed carbohydrate moderation and folic acid supplementation, while 83% (*n* = 10) recommended vitamin D assessment. However, specific targets for postprandial glucose were highly variable, with 50% (*n* = 6) favoring a 1-h target and 50% (*n* = 6) a 2-h target (or offering a choice). Notably, 0% (*n* = 0) of guidelines recommended routine supplementation with chromium, selenium, or myo-inositol for GDM treatment.

Beyond the specific recommendations, we observed variability in the recency of the included guidelines, although key bodies like ADA, Diabetes Canada, and KDA have issued recent updates [11,12,13]. The quality and type of evidence cited to support recommendations varied considerably. Core principles like overall carbohydrate management and folic acid supplementation were generally backed by higher-level evidence or well-established consensus. In contrast, more specific or contentious recommendations, particularly concerning non-routine micronutrient supplementation (e.g., chromium, selenium) and the nuances of glycemic targets or carbohydrate quality (low GI), often drew upon smaller trials, observational data, or expert opinion, likely contributing to the heterogeneity noted across different guidelines, a pattern largely maintained in recent revisions [11,12,13].

### 3.1. Macronutrient Distribution

Most guidelines recommended a macronutrient distribution generally falling within 35–50% carbohydrates, 15–20% protein, and 30–35% fats (e.g., [15,17,18,23]). The emphasis on carbohydrate control was consistent. However, variations were observed regarding carbohydrate quality. Western guidelines, such as those from the current ADA [12] and NICE [15], emphasize selecting low-glycemic index (GI) foods, fiber-rich whole grains, fruits, and vegetables to improve glycemic control, recommendations supported by evidence linking such choices to mitigated postprandial glucose excursions [24,25]. Recent ADA [12] and Diabetes Canada [13] guidelines continue this focus, increasingly suggesting individualized meal planning that may incorporate recognized healthy dietary patterns (like Mediterranean or DASH styles) adapted for pregnancy needs. The French CNGOF/SFD guideline [16] also emphasizes carbohydrate quality, advising limitation of simple sugars and preferring complex carbohydrates. In contrast, the FIGO guidance [23] and some reviewed Asian guidelines [17] placed greater relative emphasis on managing the total quantity of carbohydrates consumed. The most recent KDA guidelines [11] also emphasize individualized nutrition therapy considering preferences and culture, while generally recommending avoiding excessive simple sugars and preferring complex carbohydrates.

In addition to carbohydrate control, adequate fiber intake, typically around 20–30 g per day, was almost universally recommended to improve insulin sensitivity and glycemic control, with soluble fiber often highlighted (e.g., [11,12,13,15,16,17,23,26,27,28,29,30,31]). Some regional guidelines suggested incorporating locally available fiber sources [11,17,18].

### 3.2. Glycemic Targets

Most guidelines agreed on a fasting blood glucose target below 95 mg/dL (5.3 mmol/L) (e.g., [11,12,13,15,17,19,21,23]). However, the French guideline recommended a slightly lower target (<92 mg/dL or 5.1 mmol/L) [16]. Postprandial glucose targets exhibited significant and persistent variation, even in the latest updates from ADA [12], Diabetes Canada [13], and KDA [11]. ADA [12], Diabetes Canada [13], and KDA [11] recommend targeting either <140 mg/dL (7.8 mmol/L) at 1-h or <120 mg/dL (6.7 mmol/L) at 2-h post-meal. The French CNGOF/SFD guideline [16], along with WHO [21], focuses on a 2-h post-meal target of <120 mg/dL (6.7 mmol/L). NICE [15] uses a 1 h target of <140 mg/dL (7.8 mmol/L), while Australian/NZ guidelines reviewed [19,20] used <120 mg/dL (6.7 mmol/L) at 1 h. This lack of uniformity likely reflects differing clinical perspectives, interpretations of outcome data, and historical monitoring practices [32,33,34].

Multiple daily self-monitoring of blood glucose (SMBG), particularly postprandially, remains the standard recommendation in most guidelines [16,21,23,35,36]. Recent guidelines from ADA [12] and Diabetes Canada [13] now acknowledge the potential utility of Continuous Glucose Monitoring (CGM) for providing richer glycemic data but note the current lack of established, evidence-based GDM-specific Time-in-Range (TIR) targets, limiting its role primarily to an adjunct tool for pattern management. The KDA [11] also mentions CGM as potentially useful but similarly does not establish specific GDM targets.

### 3.3. Micronutrient Supplementation

The most significant differences across guidelines related to micronutrient supplementation beyond universally recommended folic acid and commonly advised vitamin D assessment/supplementation aligned with general pregnancy care [37,38,39]. Recommendations for other micronutrients like omega-3 fatty acids, chromium, zinc, and selenium were highly variable, with a consistent lack of endorsement for routine use in GDM management in the most recent key guidelines reviewed.

While micronutrients like chromium and zinc are theoretically linked to improved insulin sensitivity [40,41], major guidelines reviewed; including the latest from ADA [12], Diabetes Canada [13], KDA [11], and the French CNGOF/SFD [16], explicitly do not recommend their routine supplementation for managing established GDM, citing insufficient high-quality evidence. Similarly, despite research on potential benefits [42,43,44], routine omega-3 fatty acid supplementation is not endorsed for GDM management in these key North American [12,13] or European [15,16] guidelines, nor in the international [23], reviewed Asian [17,18] or the updated KDA [11] documents due to inconclusive evidence. Selenium supplementation, though hypothesized to affect glucose metabolism [45,46,47,48], was also not recommended for routine GDM management in any of the reviewed guidelines [11,12,13,15,16,17,18,19,20,23]. The discussion around myo-inositol [49] often differentiates potential preventive roles from treatment, with insufficient evidence cited for routine treatment recommendations in most major guidelines, including recent ones [11,12,13], Table 2.

## 4. Discussion

This systematic review highlights a fascinating paradox: while there is consensus around the pillars of GDM nutritional management—carbohydrate moderation, glycemic control, and selected micronutrient support—there remain significant inconsistencies in the specifics of guideline implementation across regions. These inconsistencies, persistent even in recent updates from leading organizations like ADA [12], Diabetes Canada [13], and KDA [11], reflect a complex interplay between emerging evidence, cultural dietary norms, healthcare infrastructure, and varying thresholds for clinical action. As GDM continues to rise globally, these differences warrant critical examination and potential harmonization where evidence allows.

The variability in recommendations is partly explained by the significant heterogeneity in the methodological quality of the guidelines themselves, as revealed by our AGREE II appraisal (Table 1). Guidelines developed with high methodological rigor (e.g., NICE, WHO, ADA) tend to base recommendations on formal systematic reviews and evidence grading, whereas others rely more heavily on expert consensus, leading to different conclusions even when reviewing similar evidence.

One of the most striking findings is the discrepancy in how guidelines conceptualize carbohydrate management. While most recommend 35–50% of energy from carbohydrates, the emphasis diverges. Western guidelines, particularly ADA [15] and NICE [15], stress carbohydrate quality (low GI, high fiber), supported by evidence on postprandial control [48,50]. The increasing mention of healthy dietary patterns in recent ADA [14] and Diabetes Canada [13] guidelines suggests a move towards more holistic advice. However, the practical application of GI remains challenging [51,52,53]. This difficulty stems not only from inherent food variability but also from the educational burden it places on both providers and patients, particularly in time-constrained clinical encounters or populations with lower health literacy. In contrast, some Asian guidelines [11,17,18] focus more on total carbohydrate quantity, possibly due to regional diets [54]. Yet overly restrictive approaches risk nutritional inadequacy [55]. This suggests a context-specific, food-pattern-based approach may be more pragmatic globally, although defining and validating such patterns across diverse cultures remains a significant research undertaking.

This gap between recommendation and real-world implementation is a key challenge and our AGREE II analysis highlights that few guidelines, with the exception of New Zealand’s, scored highly on “Applicability,” which assesses barriers, resource implications, and implementation tools.

The divergent glycemic targets further illustrate underlying philosophical and practical rifts in care standards. While fasting targets are relatively consistent (mostly <95 mg/dL, although the French guideline [11] suggests <92 mg/dL), postprandial goals vary widely (1 h vs. 2 h, <120 vs. <140 mg/dL) [11,12,13,16,19,21]. These differences impact clinical decisions. The TARGET trial’s finding of mixed maternal/fetal outcomes with tighter targets [56] likely contributes to the persistent lack of consensus, even in recent updates [11,12,13] and underscores the need for individualization. Discrepancies may also reflect health system capacity; achieving and managing tighter glycemic control often requires more intensive support, including frequent monitoring (potentially increasing costs associated with test strips or CGM), more readily available dietetic counseling, and quicker access to pharmacological intervention if needed. This inherently raises equity concerns, as stricter targets may be less feasible or sustainable in lower-resource settings or for patients facing significant socioeconomic barriers. The acknowledgment of CGM technology [11,12,13] adds another layer; while providing richer data, the lack of validated GDM-specific Time-in-Range targets hinders its standardized application for treatment adjustments globally. Tiered recommendations based on context [57,58] could offer a path forward, acknowledging both the ideal physiological goal and the practical realities of different healthcare environments.

Regarding micronutrient supplementation beyond folic acid and vitamin D, the consistent lack of endorsement for routine use of chromium, selenium, omega 3s, or myo-inositol for treating established GDM across major guidelines, reaffirmed in the latest updates [11,12,13] and the French CNGOF/SFD guideline [16], highlights a cautious, evidence-based approach. While research explores potential benefits [41,42,43,44,45,46,47,49], the high bar for recommending supplements during pregnancy necessitates robust RCT data, which is currently lacking for these specific agents in GDM management. Clearer distinction between prevention and treatment evidence is needed. This conservatism likely reflects the high stakes involved in prenatal care, where potential unknown risks of supplementation to the fetus must be weighed against often theoretical or poorly substantiated benefits for GDM treatment itself. This raises broader methodological questions about how guidelines should handle emerging but inconclusive evidence for potentially low-risk interventions, suggesting a need for transparent evidence grading frameworks that explicitly state the rationale for inclusion or exclusion of interventions based on the perceived balance of benefit, risk, and evidence certainty.

A profound gap remains in cultural adaptability and behavioral feasibility. Furthermore, this review highlights the general absence of recommendations tailored to racial or ethnic differences. It is well-established that the prevalence of GDM, metabolic responses to dietary patterns, and risk of complications can vary significantly across different ethnic groups (e.g., South Asian, Hispanic, Black). Future guidelines should incorporate evidence on ethnicity-specific dietary needs and responses to provide more equitable and effective care. Acknowledging these differences is a crucial step toward personalized nutrition. Few guidelines, even recent ones [11,12,13], offer detailed strategies for tailoring advice to diverse cuisines, resource limitations, or psychosocial contexts [21,23]. This often results in generic dietary advice that may be impractical, unpalatable, or culturally incongruent, leading to poor adherence and potentially suboptimal outcomes despite the guideline’s scientific validity on paper. Integrating behavioral science principles (like motivational interviewing) and practical implementation tools is crucial but underemphasized [12,15,23]. The failure to bridge this gap between recommendation and real-world application represents a major barrier to effective GDM management globally.

While this review provides a comprehensive overview, some limitations should be acknowledged. Firstly, our search was restricted to guidelines published in English or with accessible translations, which, as noted in the PRISMA flowchart, led to the exclusion of four potentially relevant documents and may have introduced a language bias. This limitation has likely contributed to the absence of guidelines from regions such as Africa and Latin America, where guidance may be published in other languages or exist as grey literature. Secondly, although we included manual searches of official websites, we may not have captured guidelines that are not formally published or indexed in major databases (i.e., “grey literature”), potentially omitting some regional or local recommendations. Finally, the included guidelines themselves vary in their methodological rigor and the quality of the evidence cited, which can affect the direct comparability of their recommendations. While we performed a formal quality appraisal using the AGREE II tool, a full risk-of-bias assessment of the primary studies within each guideline was beyond the scope of this review. These limitations, however, do not detract from the main findings but rather reinforce the call for more standardized, high-quality, and globally accessible guidelines for GDM management.

Looking forward, future guidelines must prioritize flexibility in glycemic targets, potentially using tiered approaches informed by monitoring capacity (including SMBG and CGM). Harmonization could be advanced through international collaborations, such as a global GDM task force, to develop core principles with tiered, resource-sensitive recommendations for local adaptation. A synthesized approach for clinicians can be derived from the areas of broad agreement among the reviewed guidelines. This pragmatic framework includes:

- Individualized Medical Nutrition Therapy (MNT): This is the universal cornerstone. All guidelines agree that a one-size-fits-all diet is inappropriate.
- Carbohydrate Management: Focus on quality over strict quantity. Prioritize high-fiber, low-glycemic index carbohydrates distributed across three main meals and two to three snacks to manage postprandial glucose levels. A minimum of 175 g/day is often cited to prevent ketosis.
- Glycemic Targets: Aim for a fasting glucose of <95 mg/dL (5.3 mmol/L). For postprandial targets, a consistent approach (either 1 h <140 mg/dL or 2 h < 120 mg/dL) should be chosen and applied, with individualization based on patient response and fetal growth.
- Standard Prenatal Supplementation: Continue with routine folic acid and ensure vitamin D sufficiency is assessed and treated if deficient.
- Avoidance of Non-Routine Supplements: There is a clear consensus against routinely recommending supplements like chromium, selenium, myo-inositol, or omega 3 fatty acids specifically for GDM treatment due to insufficient evidence.

Future research should focus on adequately powered RCTs evaluating the impact of these micronutrients on clinically relevant GDM treatment outcomes (e.g., need for medication, maternal glycemic control metrics, neonatal complications) in diverse pregnant populations. Explicit focus on behavioral support strategies and leveraging digital health tools should be standard. Furthermore, dedicated implementation research is required to understand how best to translate evidence-based nutritional recommendations into effective, sustainable practices within varied healthcare systems and cultural settings. Crucially, greater involvement of women with lived GDM experience in guideline development is paramount to improve relevance and applicability.

## 5. Conclusions

In conclusion, while existing nutritional guidelines for GDM provide essential principles, significant inconsistencies persist in actionable recommendations regarding carbohydrate strategies, glycemic targets, non-routine micronutrient supplementation, and practical implementation support, even considering recent updates from key organizations. These discrepancies create confusion for clinicians and inequities for patients. Moving forward requires a commitment to developing guidelines that are not only evidence-based but also flexible, culturally adaptive, behaviorally informed, and globally inclusive.

## Figures and Tables

**Table 1:** Methodological quality appraisal of included guidelines using the AGREE II instrument.

Guideline (Country/ Organization, Year)	Domain 1: Scope and Purpose	Domain 2: Stakeholder Involvement	Domain 3: Rigor of Development	Domain 4: Clarity of Presentation	Domain 5: Applicability	Domain 6: Editorial Independence	Overall Score (Domain Mean)
ADA (2024)	78%	50%	81%	100%	38%	92%	73%
Korea (2023)	83%	67%	75%	100%	46%	83%	76%
China (2022)	78%	28%	29%	100%	6%	89%	55%
NICE (2020)	89%	94%	100%	100%	100%	100%	97%
Japan (2019)	100%	56%	81%	100%	25%	100%	77%
Canada (2018)	61%	56%	71%	100%	38%	92%	70%
FIGO (2015)	83%	72%	31%	100%	63%	58%	68%
ADIPS (2014)	72%	28%	19%	83%	33%	8%	41%
New Zealand (2014)	100%	100%	88%	100%	100%	83%	95%
WHO (2013)	100%	78%	94%	100%	54%	100%	88%
France (2010)	72%	44%	23%	100%	29%	50%	53%

**Table 2:** Comparison of nutritional recommendations according to the included guidelines.

Guideline Source	Carbohydrates (%)	Protein (%)	Fat (%)	Fasting Glucose Target	Postprandial Target	Folic Acid	Vitamin D	Chromium	Selenium	Zinc	Omega-3	Myo-Inositol
ADA (USA, 2024)	40–45% (Indiv.) ^1^	15–20%	30–40%	<95 mg/dL	<140 mg/dL (2 h) OR <120 mg/dL (2 h)	1	1	0	0	0	0	0
NICE (UK, 2015)	Individualized	Individualized	Individualized	<95 mg/dL ^2^	<140 mg/dL (2 h)	1	1	0	0	0	0	0
Diabetes Canada (2023)	40–45% (Indiv.) ^1^	15–20%	30–35%	<95 mg/dL	<140 mg/dL (2 h) OR <120 mg/dL (2 h)	1	1	0	0	0	0	0
WHO (2014)	Not specified	Not specified	Not specified	<95 mg/dL ^3^	<153 mg/dL (2 h) ^3^	1	1 ^4^	0	0	0	0	0
IDF (2019)	30–50%	15–20%	30–35%	<95 mg/dL	<140 mg/dL (2 h)	1	1	0	0	0	0	0
FIGO (2015)	30–50%	15–20%	30–35%	<95 mg/dL	<140 mg/dL (2 h)	1	1	0	0	0	0	0
Japan (2019)	40–50%	15–20%	30–35%	<95 mg/dL	<120 mg/dL (2 h)	1	0	0	0	0	0	0
China (2020)	40–50%	15–20%	30–35%	<95 mg/dL	<120 mg/dL (1 h)	1	0	0	0	0	0	0
Korea (2023)	40–50% (Indiv.) ^1^	15–20%	30–40%	<95 mg/dL	<140 mg/dL (2 h) OR <120 mg/dL (2 h)	1	1	0	0	0	0	0
France (CNGOF/SFD, 2010)	Individualized	Individualized	Individualized	<92 mg/dL	<120 mg/dL (2 h)	1	1	0	0	0	0	0
Australia (ADIPS, 2014)	40–45%	15–20%	30–35%	<95 mg/dL	<120 mg/dL (1 h)	1	1	0	0	0	0	0
New Zealand (MoH, 2014)	35–50%	15–20%	30–35%	<95 mg/dL	<120 mg/dL (1 h)	1	1	0	0	0	0	0

Indiv. = Individualized based on needs, weight gain, glycemic response, and preferences. Emphasis often on carbohydrate quality (low GI, fiber) and healthy dietary patterns. 1 = Indicates recommendation for standard prenatal supplementation or assessment/treatment if deficient. 0 = Indicates no specific recommendation for routine supplementation for GDM treatment beyond standard prenatal care or dietary intake. Columns for specific micronutrients (chromium, selenium, etc.) are included to explicitly demonstrate the widespread lack of endorsement for their routine use. ^1^ Macronutrient percentages are often guides; these guidelines emphasize individualized medical nutrition therapy (MNT) focusing on carb quality and patterns. ADA recommends a minimum of 175 g carbs/day. ^2^ NICE target is <5.3 mmol/L fasting, which is equivalent to <95.4 mg/dL. ^3^ WHO 2014 targets listed are the diagnostic thresholds for GDM; the guideline itself does not specify distinct management targets. ^4^ WHO generally recommends standard prenatal care, which includes Vit D considerations, hence marked as “1”. IDF Atlas refers to general prevalence/info; FIGO provides the practical management guidance often associated with IDF internationally. Approximate ranges (~) are used where guidelines provide flexibility or focus less on strict percentages. (1 h) = 1-h postprandial; (2 h) = 2-h postprandial. The “OR” indicates either target can be used.

## Supplementary Materials

The following supporting information can be downloaded at: https://www.mdpi.com/article/10.3390/nu17142356/s1, Table S1: PRISMA checklist; Table S2: PROSPERO Protocol.

## Abbreviations

The following abbreviations are used in this manuscript:

ADA	American Diabetes Association
ADIPS	Australasian Diabetes in Pregnancy Society
APDF	Asia-Pacific Diabetes Federation
CGM	Continuous glucose monitoring
CNGOF	Collège National des Gynécologues et Obstétriciens Français (French National College of Obstetricians and Gynecologists)
DASH	Dietary Approaches to Stop Hypertension
FIGO	International Federation of Gynecology and Obstetrics
GDM	Gestational diabetes mellitus
GI	Glycemic index
IDF	International Diabetes Federation
KDA	Korean Diabetes Association
MNT	Medical nutrition therapy
NICE	National Institute for Health and Care Excellence
PRISMA	Preferred Reporting Items for Systematic Reviews and Meta-Analyses
PROSPERO	International Prospective Register of Systematic Reviews
RCT	Randomized controlled trial
SFD	Société Francophone du Diabète (French-speaking Diabetes Society)
SMBG	Self-monitoring of blood glucose
T2DM	Type 2 diabetes mellitus
TIR	Time-in-Range
WHO	World Health Organization
