# Supplementary material for: Systematic Review of Nutritional Guidelines for the Management of Gestational Diabetes Mellitus: A Global Comparison
Source: Nutrients. 2025 Jul 18;17(14):2356. doi: 10.3390/nu17142356 (PMC12300920; doi:10.3390/nu17142356)
Supplement: Supplementary file 1 [file nutrients-17-02356-s001.zip › Table S2. prospero protocol.pdf]

Systematic Review of Nutritional Guidelines for the Management of Gestational Diabetes Mellitus: A Global Comparison

PROSPERO PROTOCOL

1 Search Strategy

A comprehensive literature search was performed in PubMed, Scopus, and Web of Science to identify national and international guidelines on the nutritional management of GDM. The search terms included:

- "Gestational diabetes nutrition guidelines"
- "Dietary management of GDM"
- "GDM dietary recommendations"
  Boolean operators (AND, OR) were used to refine results. Additionally, guidelines were retrieved from official health organization websites (ADA, IDF, WHO, NICE, Diabetes Canada, Australian Diabetes Society, and Asia-Pacific Diabetes Federation).

2 Eligibility Criteria

Guidelines were included if they:

- Were published within the last 10 years.
- Were issued by national or international health organizations.
- Contained explicit dietary recommendations for GDM management.

Guidelines were excluded if they were outdated, lacked clear dietary recommendations, or were not available in English.

3 Data Extraction and Synthesis

Two independent reviewers extracted data, focusing on:

- Macronutrient distribution (carbohydrate, protein, fat intake).
- Glycemic targets (fasting and postprandial glucose levels).
- Recommendations for micronutrient supplementation.
- Behavioral counseling strategies.

Discrepancies in extracted data were resolved by a third reviewer. The information was synthesized into a comparative table highlighting differences among guidelines.

4 Risk of Bias and Quality Assessment

The guidelines were assessed using the AGREE-II (Appraisal of Guidelines for Research and Evaluation) tool to evaluate their methodological quality. Recommendations were classified based on supporting evidence, distinguishing between randomized controlled trials (RCTs), observational studies, and expert consensus.
